# Quantitative Craniofacial Analysis and Generation of Human Induced Pluripotent Stem Cells for Muenke Syndrome: A Case Report

**DOI:** 10.3390/jdb9040039

**Published:** 2021-09-22

**Authors:** Fahad K. Kidwai, Byron W. H. Mui, Konstantinia Almpani, Priyam Jani, Cyrus Keyvanfar, Kulsum Iqbal, Sriram S. Paravastu, Deepika Arora, Pamela Orzechowski, Randall K. Merling, Barbara Mallon, Vamsee D. Myneni, Moaz Ahmad, Paul Kruszka, Maximilian Muenke, Jeremiah Woodcock, Jeffrey W. Gilman, Pamela G. Robey, Janice S. Lee

**Affiliations:** 1 National Institute of Dental and Craniofacial Research, National Institutes of Health, Department of Health and Human Services, Rockville, MD 20892, USA; byron.mui@nih.gov (B.W.H.M.); nadine.almpani@nih.gov (K.A.); priyam.jani@nih.gov (P.J.); cyruskeyvanfar@gmail.com (C.K.); sriram.paravastu@nih.gov (S.S.P.); deepikaarora.23@rediffmail.com (D.A.); pamela.orzechowski@nih.gov (P.O.); randall.merling@nih.gov (R.K.M.); vamseedhar.myneni@nih.gov (V.D.M.); moaz.ahmad@nih.gov (M.A.); probey@dir.nidcr.nih.gov (P.G.R.); 2 School of Dental Medicine, Tufts University, Boston, MA 02111, USA; kulsum.Iqbal@tufts.edu; 3 Biosystems and Biomaterials Division, National Institute of Standards and Technology, Gaithersburg, MD 20899, USA; 4 NIH Stem Cell Unit, National Institute of Neurological Disorders and Stroke, National Institutes of Health, Department of Health and Human Services, Rockville, MD 20892, USA; mallonb@ninds.nih.gov; 5 National Human Genome Research Institute, National Institutes of Health, Department of Health and Human Services, Rockville, MD 20892, USA; pkruszka@genedx.com (P.K.); maxmuenke@gmail.com (M.M.); 6 Materials Measurement Laboratory, National Institute of Standards and Technology, Gaithersburg, MD 20899, USA; jeremiah.woodcock@nist.gov (J.W.); gilmanjeff@lamtec.com (J.W.G.); 7 Craniofacial Anomalies & Regeneration Section, National Institute of Dental and Craniofacial Research, National Institutes of Health, Department of Health and Human Services, Rockville, MD 20892, USA

**Keywords:** Muenke syndrome, human induced pluripotent stem cells, craniosynostosis, craniofacial abnormalities, geometric morphometric analysis

## Abstract

In this case report, we focus on Muenke syndrome (MS), a disease caused by the p.Pro250Arg variant in fibroblast growth factor receptor 3 (FGFR3) and characterized by uni- or bilateral coronal suture synostosis, macrocephaly without craniosynostosis, dysmorphic craniofacial features, and dental malocclusion. The clinical findings of MS are further complicated by variable expression of phenotypic traits and incomplete penetrance. As such, unraveling the mechanisms behind MS will require a comprehensive and systematic way of phenotyping patients to precisely identify the impact of the mutation variant on craniofacial development. To establish this framework, we quantitatively delineated the craniofacial phenotype of an individual with MS and compared this to his unaffected parents using three-dimensional cephalometric analysis of cone beam computed tomography scans and geometric morphometric analysis, in addition to an extensive clinical evaluation. Secondly, given the utility of human induced pluripotent stem cells (hiPSCs) as a patient-specific investigative tool, we also generated the first hiPSCs derived from a family trio, the proband and his unaffected parents as controls, with detailed characterization of all cell lines. This report provides a starting point for evaluating the mechanistic underpinning of the craniofacial development in MS with the goal of linking specific clinical manifestations to molecular insights gained from hiPSC-based disease modeling.

## 1. Introduction

Muenke syndrome (MS) is caused by the fibroblast growth factor receptor 3 (FGFR3) p.Pro250Arg gain-of-function mutation in the N-terminal extracellular IgII–III linker region of FGFR3, with an estimated birth prevalence of 1 in 10,000 [1,2]. It results in dramatic craniofacial deformities with no available cure. Characterized by incomplete penetrance and variable expressivity [3], MS is a rare and complex disease that remains an immense challenge to study, diagnose, and treat. Several studies have documented the qualitative clinical phenotype of MS, which includes, but is not limited to, uni- or bilateral premature fusion of one or more cranial sutures (craniosynostosis), macrocephaly without craniosynostosis, dysmorphic craniofacial features, dental malocclusion, midfacial hypoplasia, and a high arch palate [4,5,6,7,8,9,10,11,12]. However, to date, very few quantitative craniofacial morphology studies have been reported. One examined the severity of the cranial phenotype in MS infants compared to non-syndromic unicoronal synostosis [13], and a second examined the midface cephalometrics [14], but they both provided limited anthropometric data and no geometric morphometric (GMM) measurements. Still, these studies and others underscore the need to build upon quantitative craniofacial measurements to perform proper disease phenotyping, which is critically important in designing and contextualizing molecular studies [15,16].

The inaccessibility of human skeletal samples and a lack of relevant in vivo and in vitro models have hindered the investigation of MS’s pathogenic mechanisms, but MS modeling through human induced pluripotent stem cells (hiPSCs) offers alternative solutions. Rare disease research relies heavily upon modeling genetic changes and developmental pathways to recapitulate the unique aspects of human disease [17]. Twigg and colleagues reported the first MS mouse model (FGFR3 ^P244R^) [18], and it provided tremendous insight into the pathophysiological mechanisms of MS in humans; however, the heavy dependence of mouse models on the frequency of mutated alleles limits their application. The FGFR3 ^P244R^ MS mouse model also exhibits certain phenotypes that have not been previously described in humans and lacks some that have [10]. As such, the MS mouse model is an imperfect one with questionable human disease relevance depending on the tissue under examination. hiPSCs harboring the MS FGFR3 mutation are a powerful, versatile tool that overcomes many of these shortcomings. They can be reproducibly differentiated into various cell types while preserving patient-specific genetic backgrounds. These advantages allow one to construct cellularly complex and physiologically relevant disease models and to explore how variable expressivity arises. However, hiPSCs from an entire family (proband with MS and unaffected parents) have not been generated or reported previously. Doing so would create an invaluable opportunity to examine normal and disrupted craniofacial bone development and to correlate these findings with the patient’s presentation.

Taken together, there is a great need to more comprehensively describe the broad spectrum of clinical findings in MS. To that end, we first quantitatively described the craniofacial anomalies of a young male with the FGFR3 p.Pro250Arg mutation and his unaffected parents by using GMM analysis and advanced 3D imaging of the soft tissues, as well as X-rays and computed tomography (CT) scans for analysis of hard tissues. Secondly, we reported the first hiPSCs generated from a family trio, which includes a patient with MS and his unaffected parents as controls, with detailed characterization. These cell lines will serve as a reliable, patient-specific platform with which to model MS. Overall, this case report establishes a framework for systematically phenotyping families with MS and deriving hiPSCs, with the goal of linking molecular studies with disease manifestations.

## 2. Materials and Methods

### 2.1. Genetic Diagnosis

The diagnosis of Muenke syndrome was established in a proband by the identification of a heterozygous c.749C > G (p.Pro250Arg) mutation in FGFR3 by genetic testing (Advocate Medical Group, Park Ridge, IL, USA). Both biological parents were confirmed to be negative for the mutation.

### 2.2. Consents, Questionnaire, Medical History, Medication, and Dental History

Prior to any study tests, procedures, or examinations, the consent and assent forms were reviewed with and obtained from the proband and both parents. The proband and parents were enrolled onto the NIH IRB-approved protocol, 16-D-0040, Natural History of Craniofacial Anomalies and Developmental Growth Variants (PI: Lee). Information regarding past medical, surgical and dental history, and family and social history were also collected from all participants.

### 2.3. Clinical Evaluation

Clinical craniofacial analysis (dental and craniofacial dysmorphology) was performed in the Dental Clinic at the National Institutes of Health Clinical Center (Department of Health and Human Services, Rockville, MD, USA) for each participant as part of the clinical assessment. This included a comprehensive craniofacial and oral examination, anthropometric measurements of the head and face, temporomandibular joint (TMJ) exam, and assessment of cranial nerve function.

### 2.4. Photographs, X-rays, and Scans

#### 2.4.1. Photographs

Two-dimensional extraoral and intraoral photos (Canon, EOS 5D Mark III, Ota City, Tokyo, Japan) as well as 3D extraoral photos (Vectra Handheld, Canfield Scientific, Parsippany, NJ, USA) of the patients were taken at NIH Dental Clinic.

#### 2.4.2. Dental Cone Beam Computed Tomography Scans

Cone beam computed tomography (CBCT) scans were acquired using the Planmeca ProMax^®^ 3D Max (Norcross, GA, USA) at NIH Dental Clinic with the following exposure settings: normal mode, average effective patient dose 75 μSv, and 400 μm voxel size. CBCT scans were exported in DICOM format.

#### 2.4.3. Craniofacial Cephalometric Analysis

Three-dimensional reconstructions were performed in Invivo 5.4 (Anatomage, San Jose, CA, USA) and 43 craniofacial landmarks were annotated on each CBCT scan by a single researcher with expertise in 3D cephalometric analysis (KA). The 3D landmarks were selected from a larger set of 61 standardized landmarks [19]. Cephalometric analysis was also performed with the same software. Traditional 2D cephalometric measurements, including linear distances and angles, were calculated and compared to age-, sex-, and ethnicity-appropriate cephalometric norms. The list of landmarks and measurements that were included in the cephalometric analysis can be found in the supplementary material (Supplementary Tables S1 and S2).

#### 2.4.4. Geometric Morphometric Analysis

Multivariate geometric morphometric analysis was performed to explore the overall shape form variations. Landmark XYZ coordinates were exported from the In vivo software and imported into MorphoJ software [20]. For this analysis, we also implemented 3D landmark coordinate data from a large control group of healthy subjects (*n* = 191). The control group included three subgroups: an orthognathic (skeletal Class I) group (*n* = 96), a skeletal Class II (*n* = 55) group, and a skeletal Class III (*n* = 40) group. The mean age of the control group was 19.7 (8–50) years. All CBCT images in the control group were initially acquired for clinical care purposes from the University of California, San Francisco and the University of Nevada, Las Vegas through IRB-approved protocols and data-sharing agreements between the respective institutions and the NIDCR investigators (NIDCR IRB #16-D-0040, UCSF MTA #T-2014-2541; UNLV IRB #1002690-1, DSA #T-2016-3596). The scans were included in this study for secondary data analysis. All the control CBCT scans were obtained via a CB MercuRay system (Hitachi Medical Corporation, Tokyo, Japan) at 0.377 mm voxel size. The skeletal classification of the control subjects was based on the sella to nasion to A point (SNA), sella to nasion to B point (SNB), and A point to nasion to B point (ANB) angles of the included subjects, according to the normative values provided by the Steiner cephalometric analysis (SNA = 82 +/− 2, SNB = 80 +/− 2, ANB = 2 +/− 2) (Supplementary Table S3). All records were evaluated by an orthodontist (KA) and a maxillofacial surgeon (JSL), and verified for skeletal classification and diagnosis.

For the geometric morphometric analysis, Procrustes superimposition was performed without object symmetry to preserve the asymmetric effects of the craniosynostosis on craniofacial shape in the case of the proband. As our sample included subjects from different age groups, we adjusted for the effects of ontogeny by performing a multivariate linear regression of the Procrustes coordinates against subject age. We then performed a second multivariate regression on the age residuals against centroid size to control for the effects of allometry (skull size) on craniofacial shape. The residuals of these regressions were used for all subsequent analyses. A principal component analysis (PCA) was performed to examine the correlation of the overall craniofacial shape of the proband and his parents with the shape of the three normative subgroups. Because of the unequal size of the compared groups, no reliable statistical analysis could be conducted. In addition, discriminant function analysis (DFA) was used for the visualization, in more detail, of the shape differences of the subjects in comparison to the mean shape of the orthognathic (Class I) subgroup.

### 2.5. Derivation and Analysis of Human Induced Pluripotent Stem Cells

Human induced pluripotent (hiPSCs) were generated from CD34^+^ peripheral blood cells from the patients using integration-free reprogramming techniques with Sendai viral vectors (SeV, Vigene Biosciences, MD, USA) according to the manufacturer’s recommendation. Briefly, CD34^+^ cells were isolated using lymphocyte separation medium (Lonza, Basil, Switzerland) and expanded for 9 days before reprogramming. Cells were then plated on 12-well plates coated with Vitronectin XF (Nucleus Biologics, San Diego, CA, USA) in complete E8 medium (ThermoFisher Scientific, Waltham, MA, USA). Several colonies with appropriate morphology were chosen and expanded on 6-well plates.

At least three hiPSC clones were first characterized by morphology, and confirmation of the expression of the mutant allele (FGFR3 p.Pro250Arg) by MS-hiPSCs was performed by PCR amplification of FGFR3 exon 7 (Supplementary Table S4) and subsequent sequencing (Eurofins Clinical Molecular Testing Services, Louisville, KY, USA). Expression of pluripotency markers was assessed by staining for NANOG with rabbit pAb (Reprocell, Beltsville, MD, USA) and OCT3/4 with mouse mAb (Santa Cruz. Biotechnology, Dallas, TX, USA) (Supplementary Table S5). Short tandem repeat (STR) analysis to confirm the parental lineage and karyotyping to detect chromosomal abnormalities were performed by Cell Line Genetics (Cell Line Genetics, Madison, WI, USA). To assess for hiPSC differentiation capacity, teratoma assays were performed in duplicates by mixing 1 × 10^6^ cells with 50% Matrigel (Corning, Corning, NY, USA), injecting the mixture subcutaneously into NOD.Cg-*Prkdcscid Il2rgtm1Wjl*/SzJ mice (Jackson Laboratory, Bar Harbor, ME, USA), and harvesting the transplants at 8 weeks. Teratomas were cut into pieces (5 mm or less) and fixed using 4% paraformaldehyde (PFA). After embedding in paraffin, cut sections were stained using hematoxylin and eosin for identification of germ layer derivatives. NL-5 hiPSCs (source: male, CD34+ cord blood; generated at NIH Center for Regenerative Medicine (CRM); MD, USA) were used as positive controls for induced pluripotency. Please refer to cell line database source for detailed information: https://hpscreg.eu/cell-line/CRMi001-A (accessed on 22 May 2019).

Lastly, the utility of hiPSCs to model MS relies upon the fact that structural changes in FGFR3 caused by the p.Pro250Arg mutation are preserved in the newly derived mutant cell line. Therefore, screening for conformational differences between FGFR3 p.Pro250Arg and the wild-type receptor was performed using two-photon fluorescence lifetime imaging microscopy (2P-FLIM). First, cells were cultured on glass-bottom dishes and fixed using 4% PFA. A Ti-sapphire laser at 1 mW and 750 nm at 80 MHz was used to perform 2P-FLIM, and data were acquired by time-correlated single-photon counting (TCSPC) in technical replicates. Fit-free analysis was performed and represented through phasor plots and average auto-fluorescence lifetimes. A previously generated MS cell line, NIDCRi001-A, was similarly tested (data not shown). NIDCRi001-A was also extensively characterized and shown to have the identical, heterozygous FGFR3 p.Pro250Arg mutation as MS-hiPSCs [21].

## 3. Results

### 3.1. Participant One (Proband)

The proband was an 8-year-old male who presented for craniofacial evaluation and was accompanied by his parents. The proband’s gestational history was unremarkable. He was born with left unilateral craniosynostosis, right frontal bossing, and torticollis. He was confirmed to have an FGFR3 c.749C > G mutation at the age of 5 months. He was in second grade at the time of the interview, with a history of learning deficiencies and a need for speech therapy. The proband started speaking at 2.5 years of age. No hearing impairment was reported, and the patient’s vision was normal with corrective lenses. Medical history included seizures, with the last episode reported when the patient was 4 months old. He was previously treated with phenobarbital twice a day (now discontinued). No drug or food allergies were reported. The patient had undergone three surgeries without complications: (1) cranial reconstruction at the age of 7 months (he wore a helmet for 4 months following the first surgery), (2) second cranial reconstruction at the age of 1 year and 9 months, and (3) left-eye muscle reconstruction surgery. There is no family history of craniofacial anomalies or syndromes.

#### 3.1.1. Craniofacial Exam

**Figure 1:**
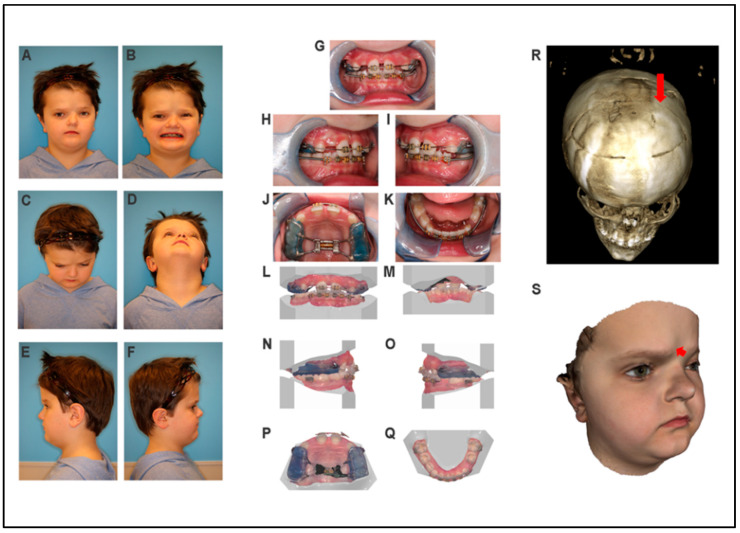
Clinical phenotype of a patient with Muenke syndrome (proband). (**A**–**F**) Two-dimensional photographs of the proband. (**G**–**K**) Intraoral photographs. (**L**–**Q**) Intraoral scan images. (**R**) Three-dimensional CBCT scan image. Red arrow shows the location of left craniosynostosis and repair. (**S**) Three-dimensional soft tissue image. Red arrow shows the frontal bossing.

*Frontal view:* the patient has a round face, with a C-shaped facial asymmetry, a broad and asymmetric forehead, and a short middle facial third (Figure 1A,B). *Eyes:* normal eye shape and size, but deep-set with mild hypertelorism and eyelid ptosis, with sparse and asymmetric eyebrows (Figure 1A). *Nose:* Small, patent nose with asymmetry and deviation to the right (Figure 1C,D). Normal nares shape with a bulbous nasal tip was observed. Snoring is reported, however, obstructive sleep apnea (OSA) has not been tested. *Lips:* the philtrum is well-defined. The patient has a thin asymmetric upper lip with downward-rotated lip commissures (Figure 1A). *Ears:* ear shape is normal with a low-set right ear (Figure 1A,E,F). *Skin:* Evidence of well-healed scalp incisions from previous cranial surgeries with no other skin lesions present. Hair has normal texture. *Neck:* No lymphadenopathy (LAD) or masses with a normal range of motion. The cranial nerve exam was normal. The TMJ was unremarkable with a normal range of motion.

*Profile:* The forehead is straight, with a deep nasal dorsum and an upturned nasal tip. Nasolabial angle is obtuse, and the midface projection and infraorbital region are flat. The mandible and chin are in a normal position, with an obtuse chin–throat angle and redundant submental tissue (Figure 1E,F). Submental view demonstrates the facial asymmetry and prominence of the right forehead (Figure 1C,D).

#### 3.1.2. Craniofacial Measurements

The head circumference is 55.2 cm, and the right and left palpebral fissures are 31 mm. The intercanthal distance is 37 mm, with an interpupillary distance of 70 mm (hypertelorism). The alar base width (ABW) is 32 mm. The philtrum length is 15 mm, and the philtrum width is 9 mm. The lip length is 17 mm. The right-ear length is 63 mm, and the left-ear length is 65 mm.

#### 3.1.3. Intraoral Examination

The patient is in mixed dentition. He has a normal eruption pattern, normal tooth morphology, no discolorations, and no congenitally missing teeth. He is undergoing orthodontic treatment, with the use of a palatal expander in combination with a face mask for the protraction of the maxilla. The expander has created a dental midline diastema (Figure 1G–Q). There are Class II molar relationships, a 4 mm overjet (5.3 mm according to cephalometric analysis), and a 20% overbite. The palate has a normal U-shape and normal rugae. There is a bifid uvula with symmetrical elevation. The tonsils are enlarged. The tongue shape and size were normal. There is normal salivary flow and the patient has good oral hygiene with healthy periodontal tissues/gingivae.

#### 3.1.4. CBCT Scan and 3D Photos

Three-dimensional photos were taken to quantify and confirm the findings of the clinical evaluation. Frontal bossing on the right side of the forehead can be observed in the 3D photos (Figure 1S).

CBCT scan results showed that several wormian bones are present near the lambda. Unilateral craniosynostosis with repair is observed on the left coronal suture (Figure 1R) with evidence of cranial osteotomies. There is a transverse osteotomy across the frontal bones and a coronal osteotomy near the coronal suture. A transverse osteotomy on the left extends posteriorly through the parietal bone as well. There are no diastases. The sutures appear fused. There is polypoid mucosal thickening in the right maxillary sinus. The left frontal sinus is not developed. Both orbits appear small, the left more so than the right. No segmentation anomalies in the visualized cervical spine.

#### 3.1.5. Cephalometric Analysis

According to the most clinically significant results of the cephalometric analysis, the skeletal relationships are Class I (ANB = 2.19°, 0.4 SDs). The most clinically significant findings included the presence of a short anterior cranial base (SN = 61.6 mm, −3.0 SDs) and midface hypoplasia (Co-A = 84.5 mm, −2.5 SDs). The mandibular base length is also significantly decreased (Go-Pog = 56.8 mm, −3.3 SDs), as is the angle of facial convexity (NA-APog = −2.61°, −2.6 SDs). In the vertical plane, both the upper (N-ANS = 40.35 mm, −3.9 SDs) and lower facial heights (ANS-Gn = 49.4 mm, −2.4 SDs) are decreased, affecting the overall facial height (N-Gn = 89.7 mm, −3.7 SDs). In the transverse plane, the facial width is increased (ZY_R-ZY_L = 109.8 mm, 3.9 SDs) and there is significant skeletal asymmetry. For a full list of the cephalometric measurements, please refer to Supplementary Table S2.

### 3.2. Participant Two (Mother)

The patient is a 46-year-old female and mother of the proband. She has a history of high blood pressure, controlled with medication (lisinopril). She has a surgical history of anterior cruciate ligament (ACL) surgery, and she is allergic to latex. There is no family history of craniofacial anomalies or syndromes.

#### 3.2.1. Craniofacial Exam

**Figure 2:**
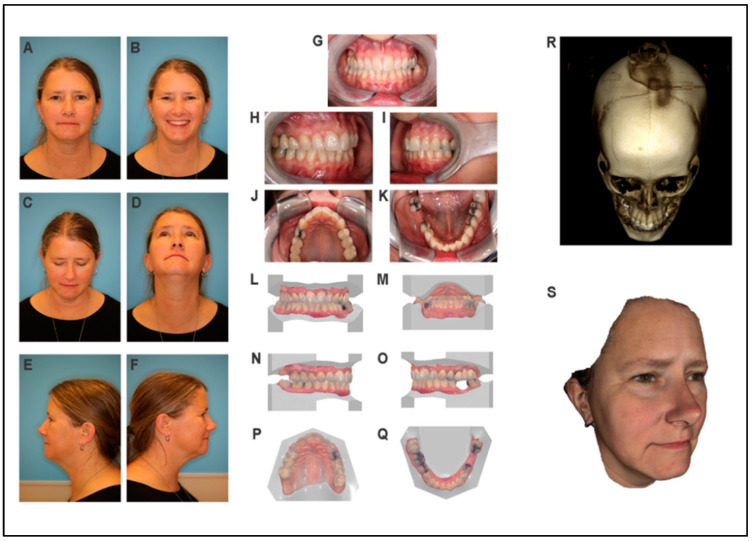
Clinical assessment of the unaffected mother of the proband. (**A**–**F**) Two-dimensional photographs of the proband’s mother. (**G**–**K**) Intraoral photographs. (**L**–**Q**) Intraoral scan images. (**R**) Three-dimensional CBCT scan image. (**S**) Three-dimensional soft tissue image.

*Frontal view:* the patient has an oval facial shape with normal facial proportions and symmetric facial features (Figure 2A,B). *Eyes:* the shape and size of the eyes are normal (Figure 2A,B). *Nose:* The nose is symmetrical and patent, with normal nares shape and nasal tip (Figure 2A,B). No snoring was reported. *Lips:* the patient has a thin symmetrical upper lip (Figure 2B). *Ears:* the ear shape and position are normal (Figure 2E,F). *Skin:* No lesions are present. Hair texture is normal. *Neck:* Normal range of motion. The cranial nerve exam was normal. *TMJ*: normal range of motion with an occasional TMJ click and pain.

*Profile*: The forehead is normal. The nose has an upturned nasal tip, and the nasolabial angle is obtuse. The midface projection is flat, whereas the mandible and chin are in normal positions (Figure 2E,F).

#### 3.2.2. Craniofacial Measurements

Head circumference: 56 cm. Right palpebral fissure: 35 mm. Left palpebral fissure: 35 mm. Intercanthal distance: 30 mm. Interpupillary distance: 60 mm. Alar base width: 37 mm. Philtrum length: 10 mm. Philtrum width: 10 mm. Lip length: 15 mm. Right-ear length: 60 mm. Left-ear length: 60 mm.

#### 3.2.3. Intraoral Exam

The patient has adult dentition, with Class I dental relationships, an overjet of 2 mm, and a 30% overbite. She has healthy oral mucosa and no intraoral lesions were detected. She has normal salivary flow and maintains good oral hygiene. The patient has a normal tongue, palate, and uvula.

#### 3.2.4. CBCT Scan and 3D Photos

CBCT scan results showed an unremarkable craniofacial skeleton (Figure 2R). There is hyperostosis of the inner table of the frontal bones. The temporomandibular joints demonstrate diminished joint space bilaterally and mild irregularity of the articular condyles. Osteophytes involve the atlanto-axial joint. The paranasal sinuses are clear. The mastoid air cells and middle ears are clear (Figure 2).

#### 3.2.5. Cephalometric Analysis

According to the most clinically significant results of the cephalometric analysis, the skeletal relationships of the mother are Class I, with a Class II tendency (ANB = 4.24°, 1.8 SDs). There is a short anterior cranial base length (SN = 64.9 mm, −3.4 SDs), a short midface length (Co-A = 77.8 mm, −3.0 SDs), and a relatively short posterior face height (S-Go = 72.43 mm, −2.0 SDs). For detailed cephalometric measurements, please refer to Supplementary Table S2.

### 3.3. Participant Three (Father)

The patient is a 45-year-old male and father of the proband. Except for a history of back problems, his medical history is unremarkable. He has no surgical history and denied any allergies. There is no family history of craniofacial anomalies or syndromes.

#### 3.3.1. Craniofacial Exam

**Figure 3:**
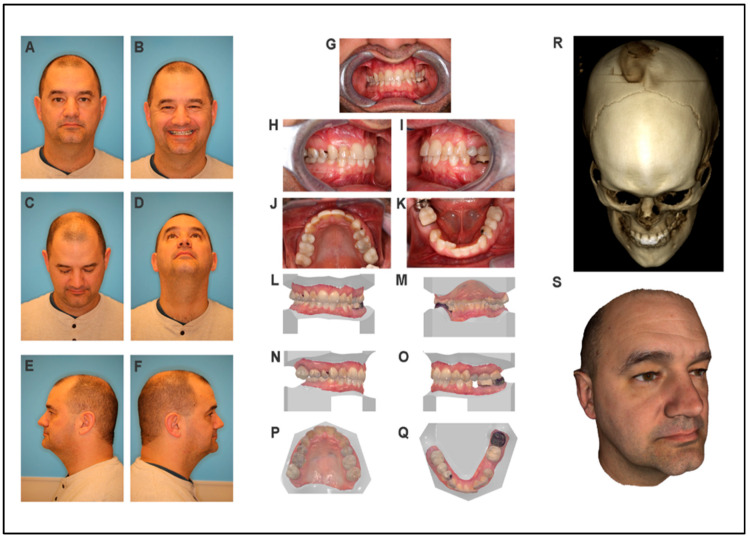
Clinical assessment of the unaffected father of the proband. (**A**–**F**) Two-dimensional photographs of the proband’s father. (**G**–**K**) Intraoral photographs (oral photo is flipped). (**L**–**Q**) Intraoral scan images. (**R**) Three-dimensional CBCT scan image. (**S**) Three-dimensional soft tissue image.

*Frontal view*: the patient has an oval facial shape, a symmetric face, and normal facial proportions (Figure 3A,B). *Eye:* the eye shape and size are normal. *Nose:* Symmetrical, normal patent nares shape with a bulbous nasal tip is noted (Figure 3C,D). No snoring is reported. *Lips:* patient has thin lips (Figure 3A,B). *Ear:* normal ear shape and position. *Skin:* No lesions are present. Hair texture is normal. *Neck:* LAD and masses are absent with a normal range of neck motion. The cranial nerve and TMJ exams were normal.

*Profile*: Straight profile with mild midface flatness and a prominent chin (Figure 3E,F).

#### 3.3.2. Craniofacial Measurements

Head circumference: 56 cm. Right palpebral fissure: 35 mm. Left palpebral fissure: 35 mm. Intercanthal distance: 30 mm. Interpupillary distance: 60 mm. ABW: 40 mm. Philtrum length: 20 mm. Philtrum width: 13 mm. Lip length: 25 mm. Right-ear length: 70 mm. Left-ear length: 70 mm.

#### 3.3.3. Intraoral Exam

The patient has adult dentition, with Class III right canine and Class I left canine dental relationships. There is a 1 mm overjet and a 50% overbite, with mild anterior dental crowding. Salivary flow is normal, and the patient maintains good oral hygiene. Normal tongue, palate, rugae, and uvula are noted.

#### 3.3.4. CBCT Scan and 3D Photos

CBCT scan results showed an unremarkable craniofacial skeleton (Figure 3R). The temporomandibular joints are unremarkable. There is mild mucosal thickening in the maxillary sinuses. The mastoid air cells and middle ears are clear (Figure 3).

#### 3.3.5. Cephalometric Analysis

Cephalometric analysis shows that the father has Class I skeletal relationships (ANB = 0.95°, −0.4 SDs), a short midface length (CoA = 84.5 mm, −2.5 SDs), a forward chin position indicated by an increased facial angle (FH-NPog = 97.7°, 2.7 SDs), and a decreased angle of facial convexity (NA-APog = −5.1°, −2.5 SDs). For detailed cephalometric analysis and measurements, please refer to Supplementary Table S2.

### 3.4. Geometric Morphometric Analysis

#### 3.4.1. Participant One (Proband)

**Figure 4:**
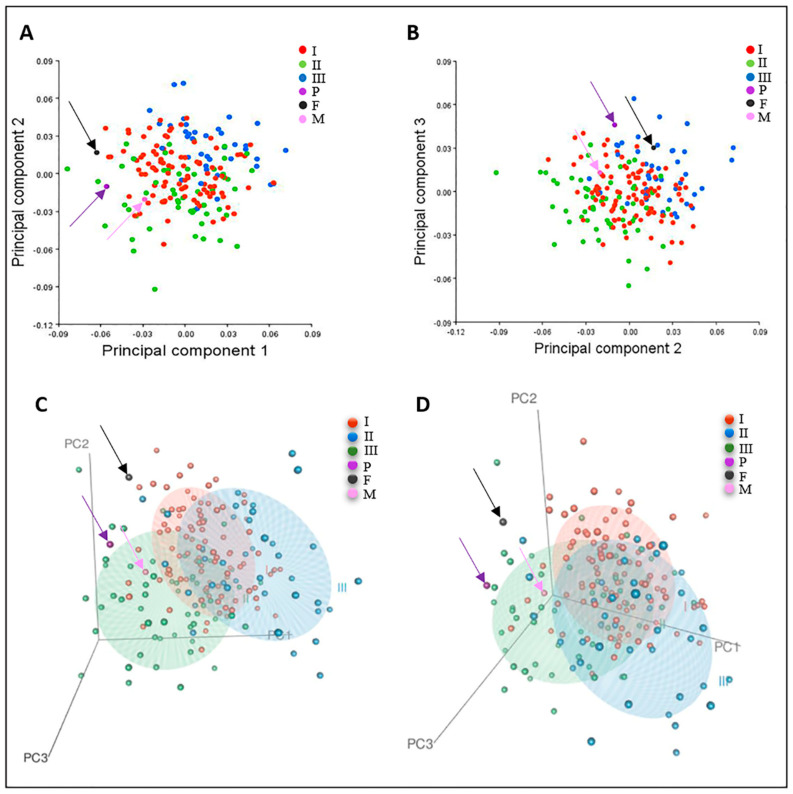
Visualization of the results of the principal component analysis (PCA). (**A**) Two-dimensional (2D) plot of the first two PC axes demonstrating the clustering of healthy subjects grouped into Class I, II and III, as well as the proband’s, the mother’s, and the father’s relative position. (**B**) Two-dimensional plot of the second and third axes of the same PCA plot. (**C**,**D**) Two different views of a three-dimensional (3D) plot of the PCA analysis including the first three PC axes and depicting the positions of the proband (P), the mother (M), and the father (F) relative to Class I, II, and III healthy subjects’ ellipsoids (I = Class I, II = Class II, III = Class III).

**Figure 5:**
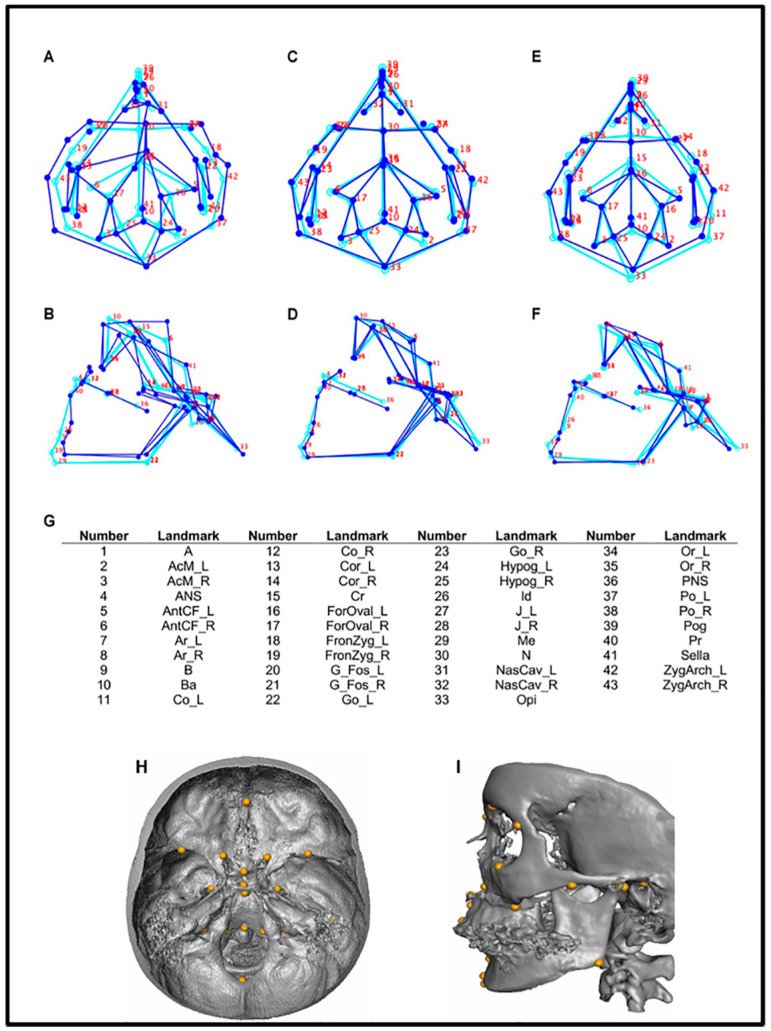
Schematics of discriminant function analysis (DFA), depicting craniofacial shape differences of the participants compared to the mean shape of the orthognathic (skeletal Class I) subgroup. (**A**,**B**) Axial and sagittal views, respectively, of the proband (dark-blue) and Class I (light-blue). (**C**,**D**) Axial and sagittal views, respectively, of the mother (dark-blue) and Class I (light-blue). (**E**,**F**) Axial and sagittal views, respectively, of the father (dark-blue) and Class I (light-blue). (**G**) A table indicating the analyzed landmark and the corresponding number on the wireframes. (**H**) Volume rendering of a 3D skull with the landmarks used in the 3D cephalometric analysis localized on a CBCT image. Each landmark is indicated by a yellow dot. Landmarks in this image are indicated from the axial view. (**I**) The 3D landmarks used in this analysis indicated from the sagittal view.

In the PCA analysis, PC1, PC2, and PC3 explain, in total, 32.3% of the craniofacial shape variance. There is, in general, an increased overlap between the control subgroups. The proband appears to be in the outer periphery of the main clusters of the normative subgroups, but relatively closer to the Class I and II clusters (Figure 4A–C). In the axial view of the DFA wireframe graphs, we can appreciate the overall degree of asymmetry of the skull of the proband (dark-blue wireframe), which also involves the cranial base (Figure 5A). In the sagittal view, we can see that the main differences are in the anterior and middle cranial base, as well as the mandible, which is more downward- and forward-positioned in comparison to the mean Class I mandibular wireframe (Figure 5B). Nevertheless, we should take into consideration the fact that the mandibular vertical position is affected by both the presence of the orthodontic appliance in his mouth and the possible indirect effect of the face mask in the vertical and sagittal position of the mandible.

#### 3.4.2. Participant Two (Mother)

In the PCA plot, the mother appears closer to Class I and III groups (Figure 4A–C). Further schematic comparison with the use of the DFA wireframes between the mother and the orthognathic control group indicates that there are some differences from the orthognathic group located mainly in the posterior cranial base, as well as the midface area, maxilla, and mandible, which appears a little more retrognathic (Figure 5C,D).

#### 3.4.3. Participant Three (Father)

In the PCA analysis plot, the father did not cluster tightly with any of the control subgroups (Figure 4A–C). However, he appeared closer to the Class I and III subgroups. Schematic comparison of the father with the orthognathic (Class I) subgroup with the use of DFA wireframes revealed some differences in the vertical position of the mandible, which appears more downward-rotated, as well as the maxilla, which looks shorter in length, and the cranial base, which differs in shape overall (Figure 5E,F).

### 3.5. Human Induced Pluripotent Stem Cell Derived from Proband and Healthy Family Members

**Figure 6:**
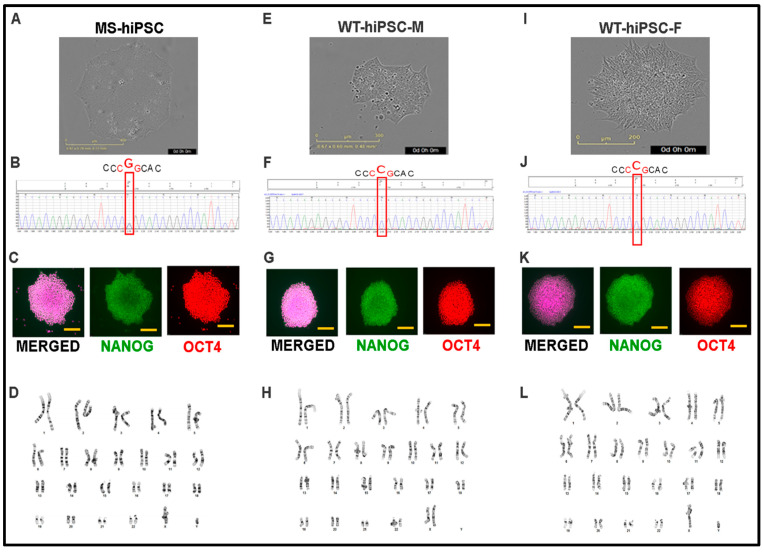
Human induced pluripotent stem cell (hiPSC) generation from a patient with Muenke syndrome (MS) and his unaffected parents. (**A**–**D**) Characterization of hiPSCs derived from a MS patient (MS-hiPSCs). (**E**–**H**) Characterization of hiPSCs derived from the mother (WT-hiPSC-M). (**I**–**L**) Characterization of hiPSCs derived from the father (WT-hiPSC-F). (**A**,**E**,**I**) Morphology of MS-hiPSCs and WT-hiPSCs. (**B**,**F**,**J**) PCR showing the FGFR3 p.Pro250Arg mutation in MS-hiPSCs and its absence in WT-hiPSCs. (**C**,**G**,**K**) Immunofluorescent staining showing expression of pluripotent markers. Non-immune immunoglobulins of the same isotypes were used as negative controls (Supplementary Table S5). (**D**,**H**,**L**) Karyotyping of MS-hiPSCs and WT-hiPSCs.

**Figure 7:**
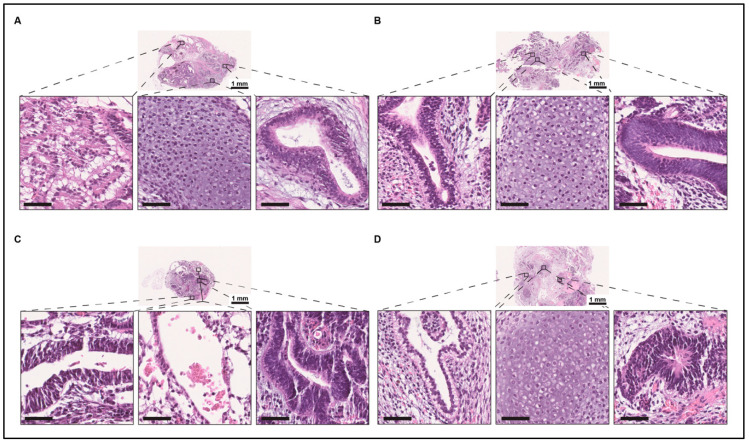
Teratoma formation assay of MS-hiPSCs, WT-hiPSC-M, and WT-hiPSC-F. (**A**) Teratoma formed by MS-hiPSCs with evidence of endodermal tubules, cartilage, and neural tubes (left to right). (**B**) Teratoma formed by WT-hiPSC-M with evidence of endodermal tubules with subnuclear vacuolization, cartilage, and neural tubes (left to right). (**C**) Teratoma formed by WT-hiPSC-F with evidence of endodermal tubules, blood vessels, and neural tubes (left to right). (**D**) Teratoma formed by the positive control NKL5 with subnuclear vacuolization, cartilage, and neural tubes (left to right). (**A**–**D**) Examples of endodermal, mesodermal, and ectodermal derivatives as proof of pluripotency (scale bar = 50 µm unless otherwise indicated).

Human induced pluripotent stem cells (hiPSCs) were generated from the proband (MS-hiPSCs) (Figure 6A–D) and the unaffected father (WT-hiPSC-F) and mother (WT-hiPSC-M) (Figure 6E–L). MS-hiPSCs and WT-hiPSCs showed typical pluripotent stem cell morphology, which includes a high nucleus-to-cytoplasm ratio; prominent nucleoli; and round, compact colonies (Figure 6A,E,I) [22]. It was confirmed that the MS-hiPSCs line had the FGFR3 p.Pro250Arg mutation (Figure 6B), whereas the WT-hiPSCs lines did not (Figure 5F,J). Expression of NANOG and OCT4 in undifferentiated MS-hiPSCs and WT-hiPSCs lines confirmed their pluripotency (Figure 6C,G,K). Karyotyping of the MS-hiPSCs and WT-hiPSCs lines showed no chromosomal abnormalities during the process of hiPSC generation (Figure 6D,H,L). Although in vitro assays of pluripotency are common and provide helpful information, the gold standard by which pluripotency is tested is by transplanting iPSCs in immunocompromised mice and identifying tissues derived from all three embryonic germ layers within the teratoma that forms. Endoderm-, mesoderm-, and ectoderm-derived tissues were readily identified using teratoma assays for all three cell lines (Figure 7A–C) with the NKL5 hiPSCs as a normal, positive control (Figure 7D).

**Figure 8:**
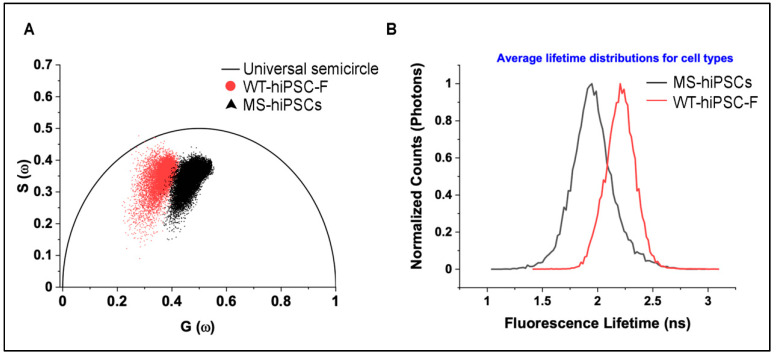
Two-photon fluorescence lifetime imaging microscopy (2P-FLIM) of MS-hiPSCs and WT-hiPSC-F. (**A**) Phasor plot of intrinsic fluorescence of MS-iPSCs compared to WT-hiPSC-F (normal control). (**B**) Average lifetime distributions for MS iPSCs and NKL5.

X-ray crystallography experiments have suggested that the p.Pro250Arg mutation results in structural changes in FGFR3, conferring greater promiscuity for atypical ligands [23,24]. In order for MS-hiPSCs to reliably mimic the human condition at the molecular level, they must, at the very least, recapitulate these receptor changes that underly its gain-of-function. As such, 2P-FLIM was used to screen for receptor differences between MS-hiPSC and wild-type FGFR3. Due to the specific characteristics of proline, such as its ring-forming backbone and lack of amide hydrogen, its replacement with arginine can cause major modifications to the protein’s overall properties [25]. Indeed, one study demonstrated that substitution of proline with alanine impacted the structural stability of a membrane protein and, in turn, its functional role [26]. These alterations can alter the autofluorescence decay of specific amino acids in the protein, such as tryptophan and phenylalanine, that can be sensitively detected by 2P-FLIM [27]. The fluorescence decay function and its time constant (fluorescence lifetime) provide direct information of a fluorophore’s type, conformation, and interaction with its local environment [28]. Results of 2P-FLIM indicated a clear difference in emission properties of the intrinsic fluorescence of MS-hiPSCs, with shorter average lifetimes in the mutant line compared to WT-hiPSC-F as a positive control (Figure 8A,B). The same trend was seen when NIDCRi001-A was analyzed (data not shown). Our results are highly suggestive that the Pro250Arg mutation results in structural changes in the receptor that modify the emission of autofluorescent proteins. Although further investigation is needed for confirmation, this simple experiment highlights the potential for MS-derived hiPSCs to serve as a platform for molecular studies.

## 4. Discussion

Due to variable expressivity and incomplete penetrance, the presentation of MS ranges from a complete absence of clinical signs to severe and life-threatening consequences of defective skull growth, such as craniosynostosis, hearing loss, and increased intracranial pressure [6]. Furthermore, many MS traits have considerable overlap with other craniosynostosis syndromes. These features highlight the need for careful and extensive evaluation of patients with MS to document the diversity in presentations. By employing qualitative and quantitative craniofacial phenotyping, our case report included deep phenotypic information of the clinical manifestations of MS that can be used to examine the clinical effects of the genetic variant. Importantly, it established a comprehensive phenotyping method that combines cephalometric and GMM analyses with advanced imaging technologies. In developmental and evolutionary biology, this strategy ultimately attempts to bridge morphological parameters with genetic and molecular data [29]. To illustrate this point, notable measurements from the patient were a shortened anterior and middle cranial base, midface hypoplasia, and differences in the mandible when compared to the Class I group. These results agree with previous work that report similar findings [8,13,30]. Additionally unique to this case report is the inclusion of both unaffected parents. The GMM and PCA demonstrate that, while clinically within the normal range, the underlying craniofacial structures suggest variability from a healthy control dataset, particularly in the region of the cranial base length and morphology. These structures, while are not clinically apparent, have downstream effects on midface and mandibular development. The threshold at which perturbations in normal development result in dysmorphology is unknown and having the data from unaffected parents begins to examine the mechanistic role of genetic variants in the proband. Interestingly, a mouse study found high expression of *FGFRIIIB* and *IIIC* isoforms in proliferating chondrocytes in the spheno-occipital synchondrosis and mandibular condyle at embryonic day 16 [31]. Situated in the cranial base, the spheno-occipital synchondrosis is a cartilaginous joint between the basioccipital and basisphenoid bones. It undergoes endochondral ossification, which relies upon a cartilage template stage, and plays a major role in the development and eventual shape of the entire head [31]. It may be possible, therefore, that the p.Pro250Arg mutation in FGFR3 exerts part of its effect on craniofacial bone development by interfering with endochondral ossification at the cranial base and downstream craniofacial structures. Further studies will be required to test this hypothesis, but this example highlights how proper morphological assessment in both affected and unaffected members in a family trio can lead to more focused research questions and examine the impact of genetic variants on craniofacial development.

An additional advantage of our phenotyping system lies in characterizing diseases with variable expressivity, due to the ability of GMM studies to quantify and perform statistically meaningful shape comparisons among subjects through pre-defined landmarks. For example, using micro-computed tomography and subsequent GMM analysis, one study quantified craniofacial differences in mice with the twisted gastrulation (*Twsg1*) mutation, which results in holoprosencephaly with incomplete penetrance and a range of defects [32]. Subsequent PCA was able to identify that narrowing of the midface explained a large proportion of the variance in mutant embryos when exposed to different doses of retinoic acid, revealing how the *Twsg1* mutation confers increased sensitivity to the teratogen. Applying a similar approach, we begin to build the framework and dataset for future GMM analyses of MS. We believe that accumulating more patient data using our method of describing MS will help characterize the clinical spectrum of the disease and inform future mechanistic studies.

Previous work has begun to unravel mechanisms responsible for MS, and MS-derived hiPSCs may help advance our understanding. FGFR3 harboring the MS mutation has been shown to have higher affinity for atypical ligands, which may cause activation of aberrant signaling pathways [23]. However, precise knowledge of the molecular pathways responsible for MS is still lacking. For the first time, therefore, we derived hiPSCs from both the patient and his parents that have undergone rigorous quality control assays to confirm the success of reprogramming. MS-hiPSCs offer a powerful in vitro method of modeling MS through directed differentiation into disease-relevant cell types. Indeed, several protocols have been established that perform differentiation of human pluripotent stem cells towards osteoprogenitors (OPs) [33,34,35]. We have also recently developed a method of lineage-specific differentiation of hiPSCs and human embryonic stem cells (hESCs) towards OPs, which allows us to generate neural-crest-derived OPs that have bone-forming capacity in vivo [36]. It has been suggested that MS has a predilection for neural crest derivatives, whereas other diseases characterized by separate FGFR3 mutations predominantly affect long bones, which are mesodermal in origin [10]. Future studies of neural-crest-derived OPs using the newly generated MS-hiPSCs can help interrogate mechanisms that result in defective bone morphogenesis and craniosynostosis. We believe this platform can provide new insights into MS pathogenesis by recapitulating the craniofacial development process. Lastly, hiPSCs reprogrammed from several patients with MS with different clinical presentations will be highly useful in identifying key gene modifiers that give rise to patient-specific phenotypes. As reprogramming workflows become more streamlined and efficient, accumulating a bank of MS-derived hiPSCs is ever more feasible.

Our case report is mainly limited by the number of patients studied, as MS is a rare and underdiagnosed disease. Consequently, we were not able to draw conclusions of any statistical significance. However, we envision that this case report will serve as a template for future studies to build upon the MS phenotype dataset. More robust GMM analyses and strength in associations can be demonstrated with a larger patient group size. This limitation further underscores the importance of developing reliable and reproducible in vitro models through the generation of hiPSCs from patients with MS.

## 5. Conclusions

In addition to qualitative clinical evaluations, this case report provides quantitative and deep phenotyping of a patient with MS and his unaffected parents using three-dimensional cephalometric and geometric morphometric analyses of craniofacial CBCT scans. We also demonstrated the successful generation of hiPSCs with the FGFR3 p.Pro250Arg mutation (MS-hiPSCs), as well as hiPSCs derived from the parents in this unique family trio. Overall, we established a comprehensive framework for assessing patients with MS and generated MS-hiPSCs as a platform to model a rare disease.

## Data Availability

The data presented in this study are available within the article and supplementary.

## Supplementary Materials

The following are available online at https://www.mdpi.com/article/10.3390/jdb9040039/s1, Table S1: List of landmarks used in the 3D cephalometric analysis and anatomical definitions. Supplementary Table S2: Showing the cephalometric analysis value of the proband, the proband’s mother and the proband’s father. Norm: Anglo-American. Supplementary Table S3: Mean values and number of standard deviations of the normal values for the general population of the angles SNA, SNB, and ANB. Supplementary Table S4: Primers and target for mutation sequencing. Supplementary Table S5: Antibodies used for immunofluorescence.
