# Supplementary material for: Quantitative Craniofacial Analysis and Generation of Human Induced Pluripotent Stem Cells for Muenke Syndrome: A Case Report
Source: J Dev Biol. 2021 Sep 22;9(4):39. doi: 10.3390/jdb9040039 (PMC8544470; doi:10.3390/jdb9040039)
Supplement: Supplementary file 1 [file jdb-09-00039-s001.zip › jdb-1355264-Supplementary .pdf]

Supplementary Table S1. List of landmarks used in the 3D cephalometric analysis and anatomical definitions.

**Landmarks**	**Symbol**	**Definition**
Nasion	N	Midpoint of the fronto-nasal suture
Orbitale	Or	Most antero-inferior point on the inferior orbital rim
Supraorbitale	SOr	The most superior and anterior point of the superior orbital rim
Frontozygomatic Suture	FronZyg	Antero-lateral point on the fronto-zygomatic suture on the orbital rim
Zygomatic Arch	ZygArch	Most latero-inferior point on the lateral curve of the zygomatic arch
Jugale	J	Deepest midpoint of the jugal process of maxilla
Nasal Cavity	NasCav	Point of junction of lateral wall of nose, nasal floor and nasal process of maxilla
A Point	A	Point of maximum concavity of the maxillary alveolar process in the midline
Prosthion	Pr	Most anterior point of the maxillary alveolar process in the midline.
Anterior Nasal Spine	ANS	Most anterior mid-point of the anterior nasal spine of maxilla
Posterior Nasal Spine	PNS	Most posterior mid-point of the posterior nasal spine of the palatine bone
B Point	B	Point of maximum concavity of the mandible alveolar process in the midline
Infradentale	Id	Point of transition from the crown/tooth of the most prominent mandibular medial incisor to the alveolar projection.
Pogonion	Pog	Most anterior point on the symphysis of the mandible
Gnathion	Gn	Point on the chin between the Menton and Pogonion.
Menton	Me	Most inferior point of the mandibular symphysis
Condylion	Co	Most superior and posterior point on the condyle of the mandible
Articulare	Ar	Most posterior point of the curvature of the head of the condyle
Gonion	Go	Highest point of the curvature of the mandibular angle formed by the junction of the ramus and the body of the mandible.
Antegonion	Ag	Highest point of the concavity of the lower border of the ramus where it joins the body of the mandible
Cribriform Plate	Cr	Most superior and mid-point of the crista galli
Midpoint of Sella turcica	Sella	Mid-point of the sella turcica
Basion	Ba	Midpoint of the anterior curvature of the foramen magnum
Opisthion	Opi	Midpoint of posterior curvature of the foramen magnum
Porion	Po	Most superior and mid-point of the external auditory meatus

Internal Acoustic Meatus	AcM	Most posterior lateral point on the posterior border of the internal acoustic meatus
Foramen Ovale	ForOval	Most antero-medial and superior point of the anterior border of the foramen ovale
Anterior Cranial Fossa	AntCF	Most anterior superior point on the anterior border of the middle cranial fossa
Glenoid Fossa	G_Fos	Most superior point of maximum curvature in the glenoid fossa
Hypoglossal Canal	Hypog	Present on the foramen magnum. Most antero-medial point of the canal. Point should roughly lie on the axis bisecting the canal in anteromedial direction.

Supplementary Table S2. Showing the cephalometric analysis value of the proband, the proband's mother and the proband's father. Norm: Anglo American.

	**Proband**			**Mother**			**Father**		
**Cranial Base Measurements**									
Anterior Cranial Base(SN) (mm)	Value	St.Dev	Dev. Norm	Value	St.Dev	Dev. Norm	Value	St.Dev	Dev. Norm
	64.9	3.0	**-1.9***	64.9	3.0	**-3.5***	71.6	3.0	**-1.9***
Posterior Cranial Base(S-Ar) (mm	29.1	4.0	-0.3	31.0	4.0	**-1.0***	34	4.0	-0.7
Saddle/Sella Angle (SN-Ar)(°)	118.1	5.0	**-1.2***	119.2	5.0	**-1.0***	123.1	5.0	-0.2
**Sagittal maxillary measurement**									
SNA (°)	81.4	3.5	-0.2	84.1	3.5	0.6	81.5	3.5	-0.1
N-A (HP)(mm)	-1.5	3.7	-0.4	-1.4	3.7	0.2	-1.4	3.7	-0.4
Midface Length (Co-A) (mm)	74.7	4.0	**-2.9***	77.8	4.0	**-3.9***	84.5	4.0	**-2.9***
Mx Unit Length (Co-ANS)	77.2	5.0	**-1.6***	80	5.0	**-2.0***	87.7	5.0	**-0.5***
**Sagittal mandibular measurement**									
SNB (°)	80.7	3.4	-0.1	80.1	3.4	-0.2	81.2	3.4	-0.1
N-Pg (HP)(mm)	-1.1	8.5	0.4	-8.6	5.1	-0.4	4.4	8.5	1.0
Md Unit Length (Co-Pog)	93.2	8.0	**-1.5***	100.0	8.0	**-1.6***	112.4	8.0	-0.1
**Sagittal jaw relationship**									
ANB (°)	0.7	1.5	-0.6	3.9	1.6	**1.5***	0.3	1.5	-0.9
Wits Appraisal (mm)	0.3	1.0	**1.3***	1.2	1.0	**2.2***	2.0	1.0	3.0
**Vertical maxillary and mandibular measurements**									
MP-SN (°)	24.3	6.0	**-1.4***	33.9	6.0	0.2	26.9	6.0	**-1.4***
Occ Plane to SN (°)	9.7	2.5	**-1.9***	13.2	2.5	-0.3	14.4	2.5	**-3.5***
FMA (MP-FH) (°)	16.7	4.5	**-2.1***	23.1	4.5	-0.2	15.1	4.5	**-2.0***
Y-Axis (SGn-SN) (°)	59.6	5.5	**-1.4***	66.2	5.5	-0.2	60.0	5.5	**-1.1***
Lower Face Hight (ANS-Me) (mm)	51.2	4.5	**-2.0***	61.3	4.5	-0.8	48.3	4.5	-0.0
LFH/TFH (ANS-Me:N-Me) (%)	56.5	3.0	0.5	56.7	3.0	0.6	55.0	3.0	-0.1

**Dentoalveolar Measurements**									
U1-SN (°)	108.1	5.5	**1.1***	104.3	5.5	0.3	118.8	5.5	**2.9***
U-Incisor Protrusion (U1-APo) (mm)	4.4	2.2	-0.7	5.3	2.2	-0.3	2.1	2.2	**-1.8***
U1-NA (°)	26.8	5.7	0.7	20.6	5.7	-0.4	37.3	5.7	**2.6***
U1-NA (mm)	5.2	2.7	0.3	2.6	2.7	-0.6	4.8	2.7	0.2
L1-MP(LADH) (mm)	31.3	3.0	**-2.9***	36.7	2.0	**-1.6***	39.2	3.0	**-2.3***
L1 Protrusion (L1-APo) (mm)	-0.8	1.7	**-2.0***	1.1	1.7	**-1.0***	-0.2	1.7	**-1.7***
L1-NB (°)	13.8	6.0	**-1.9***	21.8	6.0	-0.6	22.5	6.0	-0.5
L1-NB (mm)	0.6	4.0	**-1.9***	3.6	1.8	-0.2	2.4	1.8	-0.9*
Interincisal Angle (U1-L1) (°)	138.8	6.0	**1.5***	133.7	6.0	0.6	119.9	6.0	1.7
**Soft Tissue Measurements**									
Upper Lip to E-Plane (mm)	-5.8	2.0	**-2.2***	-9.0	2.0	**-1.5***	-10.6	2.0	**-1.3***
Lower Lip to E-Plane	-2.8	2.0	0.4	-5.5	2.0	**-1.7***	-7.4	2.0	**-2.7***
Nasolabial Angle (Col-Sn-UL) (°)	104.3	8.0	0.3	127.0	8.0	**3.1***	111.8	8.0	**1.2***
Chin Angle (Ig-Pg-MP) (°)	70.5	5.0	0.1	69.9	5.0	0.0	60.5	5.0	**1.9***

Abbreviations: ** $p < 0.05$*

Supplementary Table S3. Mean values and number of standard deviations of the normal values for the general population of the angles SNA, SNB and ANB, which were used for the classification of the dentoskeletal malocclusions for the normative control groups.

	Groups	SNA	SNB	ANB
**Mean values**	Class I	83.10317	79.73317	3.37
	Class II	82.685	77.035	5.65
	Class III	79.72636	82.46591	-2.73955
**No. of Standard Deviations from the Norm Value**	Class I	3.926698	3.929718	1.126383
	Class II	3.762325	3.450656	2.103371
	Class III	4.734917	5.663599	2.249172

Supplementary Table S4. Primer and target for mutation sequencing

	**Target**	**Forward/Reverse primer (5'-3')**
**Mutation sequencing**	*FGFR3* (c.749C>G), 191bp	CGGCAGTGGCGGTGGTGGTGA<br>GACCCAAATCCTCACGCAACC<br>GenBank Accession #NG_012632.1

Supplementary Table S5. Antibodies used for immunofluorescence

Protein	Antibody	Catalog #	Supplier
Primary Antibody Anti-NANOG	Anti-Human; Rabbit polyclonal IgG	RCAB004P-F	Reprocell (distributed by CosmoBioUSA)
Primary Antibody Anti-OCT3/4	Anti-Human; Mouse monoclonal IgG2b	SC-5279	Santa Cruz
DAPI	Anti-Human	Ab228549	abcam
Secondary antibody Alexa Fluor 488	Goat anti-rabbit IgG	A-11008	Invitrogen
Secondary antibody Alexa Fluor 594	Goat anti-mouse IgG	A-11005	Invitrogen
Alexa Fluor® 488-conjugated Isotype Control	Anti-Human; Rabbit polyclonal IgG	IC1051G	R&D Systems
Alexa Fluor® 594-conjugated Isotype Control	Anti-Human; mouse monoclonal IgG	IC0041T	R&D Systems
